# The impact of perceived discrimination on depressive symptoms and the role of differentiated social support among immigrant populations in South Korea

**DOI:** 10.1186/s12939-019-0910-9

**Published:** 2019-01-11

**Authors:** Chaelin Karen Ra, Jimi Huh, Brian Karl Finch, Youngtae Cho

**Affiliations:** 1 Department of Preventive Medicine, University of Southern California Keck School of Medicine, Los Angeles, CA USA; 2 Department of Sociology and Spatial Sciences, Center for Economic and Social Research, University of Southern California Dornsife College of Letters, Arts and Sciences, Los Angeles, CA USA; 3 Department of Health Science and Services, School of Public Health Seoul National University, 1 Kwanak-ro, Kwanak-gu, Seoul 151-742 South Korea

**Keywords:** Discrimination, Social support, Depressive symptoms, Immigrants, South Korea

## Abstract

**Background:**

Previous studies demonstrated a positive association between perceived discrimination and mental health problems among immigrants in countries that traditionally host immigrants. Recent trends in international migration show that there has been a significant increase in immigrant populations in East Asian countries. These newer host countries have different social contexts from traditional ones, yet mental health among these immigrants and its relationship to discrimination are under-researched. Thus, this study aimed to examine the association between perceived discrimination and depressive symptoms among immigrants in one of the newer host countries, South Korea. Moreover, we investigated if differentiated social support (ethnic, host or other support) serves as a moderator of discrimination for depressive symptoms.

**Methods:**

This study used survey data from the 2012 Korean Social Survey on Foreign Residents (*N* = 1068), restricted to adults 20 years or older. Multiple linear regression models were conducted to estimate the association between perceived discrimination, social support, and depressive symptoms among immigrants in South Korea.

**Results:**

Perceived discrimination showed a strong positive association with depressive symptoms among immigrants, and ethnic and host support was directly positively associated with depressive symptoms. Furthermore, ethnic support moderated the effects of perceived discrimination on depressive symptoms.

**Conclusion:**

Community-level interventions providing immigrants opportunities to increase social networking members from the same country as well as the native-born in a host country may be helpful resources for improving mental health among immigrants in South Korea. Also, raising awareness of racial discrimination among members in South Korea would be crucial.

## Background

Immigration is a stressful life event where immigrants have to adapt to a new environment [1]. Immigrants face tremendous difficulties such as language barrier, loss of important social ties, acculturative stressors, and racial discrimination during the migration process [2]. They perceive racial discrimination due to their experiences of unfair and ostracizing treatment of their own ethnic groups in a host country [3]. According to the stress model, perceived discrimination provokes strong negative emotions and generates psychological distress [3]. This can affect biological processes and in turn, negatively impact mental and physical health [3]. An immigrant may perceive himself or herself being discriminated by members of a host country, and this can be one of many stressors which contributes to poor mental health including depressive symptoms [2, 4]. The association between perceived discrimination and poor mental health outcomes among immigrants has been well documented in several countries that have traditionally hosted immigrants [3]. Among Asian Americans, studies have been shown to associate perceived discrimination with depressive symptoms [5–9]. A more recent study in Taiwan, one of the newer host countries in Asia, also reported such finding [10].

At the same time, a growing body of research has demonstrated the role of protective factors, such as emotional support, as resources for coping with stress in immigrant populations [11]. Social support has been identified as one of the key protective factors for immigrant mental health. Social support refers to emotional, informational, and instrumental supportive functions that significant others (e.g. family members and friends) perform for the individual [11]. When immigrants leave their home countries, they lose essential social ties around which they structure their lives [12, 13]. They face the challenges of re-creating their social support system in a new environment [12]. During this process, social networks are transformed, and such changes may lessen emotional support [12]. A number of similar studies demonstrated the importance of social support during migration [14–16]. To evaluate the effectiveness of social support as a resource, two models are assessed – 1) the main effect model and 2) the stress-buffering model [14, 17]. The main effect model implies that social support is directly associated with immigrants’ mental health, controlling for perceived discrimination [14]. In the stress-buffering model, social support protects mental health through buffering the effect of perceived discrimination [17]. The main effect model of social support on mental health is strongly supported [2, 11, 12]. For example, higher social support has been related to lower levels of depressive symptoms [11]. However, depending on the particular immigrant group studied, the findings of the stress-buffering effect in the relationship between perceived discrimination and mental health were inconsistent in previous research [2, 6, 11, 17–20].

An immigrant’s family and ethnic community provide the initial and crucial social support to protect his or her mental health [2]. However, if the host society is not receptive to immigrants, it may not have a presence within the support network [2, 12, 18, 21–23]. Host support is also known as a protective factor for immigrants. Interaction with members of the host country can help immigrants not only learn the language and new roles, but also adapt to the new culture easier [12]. Moreover, having native connections in the support network helps the immigrant feel more comfortable in the new environment [24]. Jasinskaja-Lahti and colleagues found that more interaction with host support networks is associated to less psychological stress symptoms [2]. Active social contacts with host support networks showed a protective effect on the mental health among immigrants who had experienced discrimination in the host society [2].

On the other hand, living in ethnic enclaves is thought to be protective of immigrant health [25], while increased contact with individuals native to the host country, has been shown to be associated with increased acculturative stress and discrimination [26]. Because of this, association with members of the host country can be seen as a double-edged sword for immigrants; positive social interactions with members of a host country are essential for cultural adaptation and health, while discriminatory interactions are deleterious.

Previous studies have addressed the relationship of perceived discrimination, social support and mental health among immigrants in traditional host countries such as the United States. However, recent trends in international migration show that there has been a significant increase in the immigrant population in East Asian countries such as Japan, Taiwan, and South Korea. Most significantly in South Korea, the rate of increase in the foreign-born population between 2001 and 2011 [27] was around 55%, which is much higher than the average of the Organization for Economic Co-operation and Development (OECD) countries (23%) whilst its change in the United States was less than 20% [27]. As of 2015, more than 1.6 million immigrants resided in South Korea, which is nearly a 30 fold increase from the last 20 years (49,500 as of 1990) [28]. These trends remain strong and are likely to grow – the proportion of foreign residents in South Korea is projected to reach 5% of the total population by 2020 [4].

These newer host countries such as South Korea have different social contexts from traditional host countries such as the United States. Koreans have a collectivist cultural orientation [29], and South Korea is a homogeneous society with 97% of its population consisting of ethnic Koreans [4]. In terms of racial discrimination, there exists positive discrimination towards people with whiter skin from European countries while those with darker skin living in Asia, Africa, and Latin America are more likely to be discriminated against by many Koreans [30]. Also, it has a relatively short history of immigration. 1990 was the first year of recording the number of foreign residents [4]. The composition of immigrant populations is also different as South Korea has much less diverse immigrant populations compared to traditional host countries. Immigrants in South Korea are generally classified into permanent residents and naturalized citizens. The Korean government started to grant permanent residency to those who stayed in Korea with an F-2 status (residence visa) for more than 5 years in 2002 [4]. The history of naturalized citizens is relatively short as well. 1957 was the first time a pure foreigner acquired Korean nationality [4]. Foreigners who became naturalized citizens were fewer than 100 persons per year until 1990 [4]. The number of naturalized citizens increased along with the rapid increase of foreign residents in the 1990s [4]. The majority of them came to South Korea as marriage migrants or labor migrants. Most of the permanent residents are from other East Asian countries, including China (the Korean Chinese), Taiwan and Japan, whereas most of the naturalized citizens are from China (the Korean Chinese), Vietnam and other Southeast Asian countries. Marriage migrants constitute about 50% of permanent residents and 60% of naturalized citizens whereas labor migrants constitute approximately 27% of permanent residents and 8% of naturalized citizens [28].

Immigrant mental health in newer host countries such as South Korea is important from both human rights and social equity-based perspectives. However, studies of immigrant health in newer host countries are still underway, and existing studies focus on certain types of immigrants such as labor migrants, or focus on physical health in geographically limited areas [31]. In addition, previous surveys for immigrants in South Korea are considered to be unsystematic and inconsistent as they have been performed by various government agencies or research centers to address short-term needs and goals [4]. As it stands, perceived discrimination, differentiated social support and immigrant mental health in South Korea are poorly understood. Thus, the findings of this study would have potential implications to inform future interventions, and provide additional evidence for policy makers who seek to decrease the impact of risk factors and improve the health of immigrant population. Moreover, the findings of this study would also contribute to the realization of the United Nations health-related Sustainable Development Goals, which aim to ensure healthy lives and promote well-being at all ages by addressing one of the current mental health issues and possible risk and protective factors related to it among underserved population in South Korea.

More research is needed to examine perceived discrimination and immigrant’s mental health among those newer host countries. Thus, this study aimed to examine the association between perceived discrimination and depressive symptoms among immigrants in one of the newer host countries — South Korea. Moreover, we also investigated whether 1) general social support moderates the effects of perceived discrimination on depressive symptoms, and 2) the role of various sources of social support (e.g. ethnic and host) in the association between perceived discrimination depressive symptoms.

## Methods

### Data

This data was collected by the IOM Migration Research & Training Center of Korea (IOM MRTC). Immigrant population lists provided by the Ministry of Justice were used to draw the sample through the stratified random sampling. Data collection teams reached out to participants, and paper-pencil surveys were administered. The survey was translated into 7 languages (Chinese, Japanese, Vietnamese, English, Thai, Cambodian and Tagalog). More information about the survey design and methods can be found at the IOM MRTC website (http://www.iom-mrtc.org/). This research received IRB exemption from the IRB committee at Seoul National University since its regulations clarified that studies using government administered publicly available secondary data were exempt from the review. The sample for this analysis was restricted to those who responded to perceived discrimination and depressive symptom questionnaires. We used listwise deletion to manage missing data values [32]; this yielded a final sample size of 1068.

### Measures

#### Outcome

The depressive symptoms were assessed by the modified version of the Hopkins Symptom Checklist (HSCL) [33, 34]. Depressive symptoms were one of three subscales and were measured by seven items: thoughts of ending your life, poor appetite, crying easily, feeling lonely, feeling blue, feeling no interest in things, and feeling hopeless. Respondents were instructed to select one of five choices to indicate the frequency with which they experienced each symptom over the past month (1-Never, 2-rarely, 3-sometimes, 4-often, 5-very often). The sum scores of seven items were used as a “depressive symptoms” outcome (score range: 7–35).

#### Predictors

Perceived discrimination was assessed with the following question: “Have you ever experienced racial discrimination?” This question had binary response categories: yes (1) or no (0). Social support was assessed by asking respondents the following question: “Do you have anyone who you can talk to when you are worried?” This question allowed participants to choose all that apply and had four response categories: (1) yes – from the same country, (2) yes – from South Korea, (3) yes – from another country (besides the same country and South Korea), (4) no. Four variables were generated from the response – (1) ethnic support, (2) host support, (3) other support, (4) general support (no vs. any support ((1–3) combined)) (Table 2).

#### Covariates

Acculturation was assessed by two measures – Korean language fluency scored from 1 (poor) to 5 (excellent) for four categories (speaking, listening, reading and writing) and years lived in Korea. Country of origin was categorized into 3 groups – Western countries (e.g. the United States or Canada), East Asia (China, Japan and Taiwan) and South Asia (e.g. Vietnam, Thailand, the Philippines and Cambodia). This classification followed a conventional way of differentiating ethnic groups in South Korea - Northeast Asians, Southeast Asians, Middle-East, African and Western countries. Since the immigrants of Middle-East and African populations were very small, they were not sampled in the survey. Thus, those three ethnic groups were used. Age was classified into 5 categories (under 30, 30–39, 40–49, 50–59, 60 or older). Visa type was divided into the following categories: ‘F-2/F-6 - residents, ‘H2/F-4 – extension of sojourn period for overseas Koreans and working visit, and ‘other’. Current marital status was recorded as ‘never married’, ‘currently married’ and ‘other’. Educational attainment was divided into the following categories: ‘middle school or less’, ‘high school graduation’, ‘some college’ and ‘university or more’. Household income was recorded as one of five categories (under $1000, $1000-2000, $2000-3000, $3000-4000, and over $4000).

### Data analysis

Descriptive analyses were reported in Table 1. Association of key predictors - perceived discrimination, general social support, and three differentiated social support (ethnic, host and other) with depressive symptoms as well as frequencies and proportions of key predictors were tested next. Statistical analyses were performed using SAS v9.4 [35] to conduct multiple linear regression to investigate the association between perceived discrimination, differentiated social support, and depressive symptoms adjusted for sex, age, country of origin, type of visa, education level, income level, marital status and Korean proficiency. In the first set of models, perceived discrimination and general social support were used as main regressors, then the interaction term of the main regressors was included. The second set of models used the more detailed measures of social support: ethnic, host, and other, with the same interactions specified as above.

**Table 1:** Sociodemographic Characteristics of the Analytic Sample

	Number	Percent
Country of origin^a^		
Western countries	40	3.75
East Asia	809	75.75
South Asia	219	20.51
Age		
Under 30	238	22.28
30–39	295	27.62
40–49	304	28.46
50–59	174	16.29
60 or over	57	5.34
Visa type^b^		
F-2/F-6	641	60.02
H-2/F-4	256	23.97
Other	171	16.01
Sex		
Male	286	26.78
Female	782	73.22
Current Marital Status		
Currently married	910	85.21
Never married	75	7.02
Other	83	7.77
Household income^c^		
Under $1000	139	13.01
$1000–2000	374	35.02
$2000–3000	286	26.78
$3000–4000	122	11.42
Over $4000	147	13.76
Educational attainment		
Middle school or less	334	31.27
High school graduation	424	39.70
College	117	10.96
University or more	193	18.07
Acculturation		
Korean Language Fluency(M/SD)^d,e^	15.08	3.98
Years lived in Korea(M/SD)^e^	10.58	10.73
Unweighted N	1068	

Abbreviations: *N* Sample size, *M* mean, *SD* Standard Deviation

Note. ^a^ Country of Origin – Western countries: The United States and Canada; East Asia: China, Japan and Taiwan; South Asia: Vietnam, Thailand, the Philippines and Cambodia. ^b^ Visa Type: F-2: Residence visa, F-6: Foreign spouse, F-4: Extension of sojourn period for Overseas Koreans, H-2: Working visit ^c^ US dollars. ^d^ Score range: 1 (poor) to 5 (excellent) for four categories (speaking, listening, reading and writing). ^e^ Continuous scores were centered zero

## Results

Table 1 reports descriptive statistics of sociodemographic characteristics for the analytic sample of participants (*N* = 1068). The majority of immigrants in the sample were from East Asia (75.75%). Most participants were relatively young (78.36% were younger than 50 years old), and there were no notable differences in the age structures. Of 1068 participants, 641 (60.02%) were under a residence visa or foreign spouse visa (F-2/F-6). The female proportion was much higher (73.22%) and so was the proportion of currently married (85.21%). Most were educated less than college level (31.27% for middle school or less and 39.7% for high school graduation level). The average years lived in Korea was 10.58 years.

Table 2 includes descriptive statistics of key variables and their correlations with depressive symptoms. Of 1068 participants, 773 (72.38%) reported that they experienced racial discrimination at least once, and it was significantly related to depressive symptoms (*p* < .001). Most participants indicated that they have at least one person to talk to (92.04%), and it was negatively related to depressive symptoms (p < .001). Among the three social support segments, ethnic support was the highest (58.71%), and ethnic and host supports were significantly and negatively related to depressive symptoms (*p* < .001).

**Table 2:** Frequencies, probabilities of key predictors and their correlations to depressive symptoms (*N* = 1068)

	N	%	Depressive symptoms	
			β	*P value*
Perceived discrimination				
Never	295	27.62	1.84	<.001
At least once	773	72.38		
General social support^a^				
None	85	7.96	−2.18	<.001
One or more	983	92.04		
Ethnic support				
None	441	41.29	−1.00	<.001
One or more	627	58.71		
Host support				
None	549	51.4	−1.61	<.001
One or more	519	48.6		
Other support				
None	1038	97.19	−0.69	0.4
One or more	30	2.81		

Abbreviations: *N* Sample size

^a^ General social support included three segmented supports (ethnic, host and other)

Table 3 provides the linear regression coefficients for depressive symptoms. In Model 1, perceived discrimination was positively associated with depressive symptoms, and it was statistically significant (β = 1.98, at *p* < .0001). Meanwhile, general support was significantly negatively associated with depressive symptoms (β = − 2.05, *p* < .0001). In Model 2, the interaction term (discrimination by general support) was significant (β = − 2.11, *p* = .04), such that the effect of discrimination was significantly dampened for those with higher levels of social support, relative to those with lower levels of social support.

**Table 3:** Association of general support with depressive symptoms among immigrants in South Korea (*N* = 1068)

	Depressive Symptoms			
	Model 1^a^		Model 2^b^	
	β	*P* value	β	*P* value
Intercept	12.23	<.001	10.87	<.001
Discrimination	1.98	<.001	3.91	<.001
General support	−2.05	<.001	−0.59	0.50
Discrimination X general support			−2.11	0.04
Sex				
Male	–			
Female	1.49	<.001	1.44	<.001
Age				
Under 30	–			
30–39	− 1.06	0.01	− 1.04	0.01
40–49	− 1.34	<.001	− 1.34	<.001
50–59	− 1.86	<.001	−1.87	<.001
60 or over	−1.55	0.04	−1.55	0.04
Country of origin^c^				
Western countries	–			
East Asia	−0.47	0.54	− 0.42	0.59
South Asia	−0.05	0.96	0.01	0.99
Visa type^d^				
F-2/F-6	–			
H-2/F-4	−0.33	0.40	− 0.34	0.39
Other	−0.76	0.07	−0.76	0.07
Current Marital Status				
Currently married	–			
Never married	−0.09	0.89	−0.04	0.95
Other	1.34	0.01	1.37	0.01
Household income^e^				
Under $1000	–			
$1000–2000	0.14	0.75	0.13	0.76
$2000–3000	− 0.83	0.07	− 0.83	0.07
$3000–4000	− 0.58	0.29	− 0.57	0.30
Over $4000	−1.45	0.01	−1.44	0.01
Educational attainment				
Middle school or less	–			
High school graduation	0.33	0.31	0.36	0.27
College	0.73	0.12	0.75	0.11
University or more	0.68	0.14	0.68	0.14
Acculturation				
Korean Language Fluency^f^	−0.05	0.13	−0.05	0.15
Years lived in Korea^f^	0.01	0.63	0.01	0.66

Note: ^a^ Included perceived discrimination and general social support as main regressors. ^b^ Included perceived discrimination, general social support and the interaction term of discrimination and general social support as main regressors. ^c^ Country of Origin – Western countries: The United States and Canada; East Asia: China, Japan and Taiwan; South Asia: Vietnam, Thailand, the Philippines and Cambodia. ^d^ Visa Type: F-2: Residence visa, F-6: Foreign spouse, F-4: Extension of sojourn period for Overseas Koreans, H-2: Working visit ^c^ US dollars. ^e^ US dollars. ^f^ Continuous scores were centered zero

Table 4 shows the linear regression coefficients for the depressive symptoms when three different types of supports were included instead of only general support. In Model 1, the main effect of ethnic and host supports were significant for depressive symptoms (β = − 0.74, *p* = .02; β = − 1.47, *p* < .001 respectively). In Model 2, we found that only ethnic support significantly moderated the effect of perceived discrimination (β = − 1.49, *p* = .02), as in those with no ethnic support experience large discrimination effects, while those with ethnic support experience negligible, or at least, less discrimination effects.

**Table 4:** Association of segmented support with depressive symptoms among immigrants in South Korea (*N* = 1068)

	Depressive Symptoms			
	Model 1^a^		Model 2^b^	
	β	*P* value	β	*P* value
Intercept	11.79	<.001	11.29	<.001
Discrimination	1.88	<.001	3.04	<.001
Ethnic support	−0.74	0.02	0.30	0.6
Host support	−1.47	<.001	−1.10	0.05
Other support	−0.98	0.23	0.25	0.88
Discrimination X ethnic support			−1.49	0.02
Discrimination X host support			−0.55	0.40
Discrimination X other support			−1.74	0.35
Sex				
Male	–			
Female	1.51	<.001	1.46	<.001
Age				
Under 30	–			
30–39	−1.02	0.02	−1.02	0.02
40–49	−1.29	0.01	−1.28	0.01
50–59	−1.83	<.001	−1.84	<.001
60 or over	−1.45	0.06	−1.48	0.05
Country of origin^c^				
Western countries	–			
East Asia	−0.67	0.40	−0.92	0.26
South Asia	−0.20	0.82	−0.41	0.65
Visa type^d^				
F-2/F-6	–			
H-2/F-4	−0.51	0.19	−0.53	0.17
Other	−0.87	0.04	−0.92	0.03
Current Marital Status				
Currently married	–			
Never married	0.01	0.99	0.12	0.86
Other	1.27	0.01	1.32	0.01
Household income^e^				
Under $1000	–			
$1000–2000	0.11	0.80	0.08	0.85
$2000–3000	−0.87	0.06	−0.85	0.06
$3000–4000	− 0.58	0.29	− 0.60	0.27
Over $4000	−1.37	0.01	−1.42	0.01
Educational attainment				
Middle school or less	–			
High school graduation	0.33	0.31	0.34	0.30
College	0.66	0.16	0.65	0.17
University or more	0.79	0.09	0.78	0.09
Acculturation				
Korean Language Fluency^f^	−0.04	0.22	−0.04	0.25
Years lived in Korea^f^	0.01	0.58	0.01	0.66

Note: ^a^ Included perceived discrimination and general social support as main regressors. ^b^ Included perceived discrimination, general social support and the interaction term of discrimination and general social support as main regressors. ^c^ Country of Origin – Western countries: The United States and Canada; East Asia: China, Japan and Taiwan; South Asia: Vietnam, Thailand, the Philippines and Cambodia. ^d^ Visa Type: F-2: Residence visa, F-6: Foreign spouse, F-4: Extension of sojourn period for Overseas Koreans, H-2: Working visit ^c^ US dollars. ^e^ US dollars. ^f^ Continuous scores were centered zero

## Discussion

This is the first study to explore the links between perceived discrimination, social support and depressive symptoms in South Korea, a new host country in Asia. Furthermore, this study investigated the main and moderating effects of not only general support but also differentiated social support related to perceived discrimination and depressive symptoms. The findings of this study partially support the main effect model and the stress-buffering model. The results demonstrated that perceived discrimination was positively associated with depressive symptoms. Any social support was independently adversely associated with depressive symptoms. General social support attenuated the impact of perceived discrimination on depressive symptoms. Interestingly ethnic support moderated the association between perceived discrimination and depressive symptoms. However, host support did not moderate the negative effects of perceived discrimination on depressive symptoms.

Consistent with previous findings, perceived discrimination was positively associated with depressive symptoms among immigrants in South Korea. Immigrants face racial discrimination in a host country, which may cause strong negative emotions and generate psychological distress [3]. This can affect biological processes and in turn, negatively impact mental health [3]. That is, an immigrant may perceive himself or herself being discriminated by members of a host country, and this can be one of stressors which contributes to poor mental health including depressive symptoms [2, 4].

At first glance, we found that general social support for perceived discrimination would contribute to reducing depressive symptoms. This finding is consistent with previous studies conducted on Latino immigrants, African-Americans and Asian Americans in traditional host countries such as the United States, Canada, and the United Kingdom [2, 6, 11, 17–20]. Social support in general can provide protection from effects of stressors like discrimination among immigrants [21, 36]. Social support protects the negative effects of perceived discrimination by providing both practical resources and emotional support (e.g., expression of love, empathy, concern) [37]. Most immigrants go through significant changes in their lives during the acculturation process [7] including separation, feeling of loss, and isolation to a new cultural environment [38]. The emotional burden of adapting to a new culture can bring about acculturative stress [39] and experiencing negative treatment from the host population [40]. Thus, it is not difficult to infer that having emotional support from someone plays a very important role in immigrants’ depressive symptoms when they lose their original support from their home country and experience discrimination in South Korea.

The results also provide strong evidence for the importance of emotional support from the same co-ethnic immigrants in South Korea. Ethnic support not only was associated with depressive symptoms but also moderated the effect of perceived discrimination on the depressive symptoms. The majority of previous studies found either no moderating effect or even a negative impact of ethnic support on depressive symptoms. For instance, a study in Finland [2] indicated that immigrants who perceived more discrimination may try not to appear ethnic behavior, and may even stop contacting with their ethnic community to avoid further discrimination.

However, in this study, there was a strong protective effect of ethnic support between perceived discrimination and depressive symptoms. Why is this different from previous findings? Even though the number of foreign residents has dramatically increased for the last two decades, it is a relatively new phenomenon in South Korea. Thus, there is currently few community organizations and social service providers in South Korea that are not well established. On the other hand, compared to traditional host countries, immigrants in South Korea are mostly marriage migrants or foreign labors, who are likely to come to South Korea without their family members. In the previous studies, family support among immigrants was a primary source of the sociopsychological process of dealing with discrimination among Asian immigrants in the United States [17, 41]. Without family support, immigrants may seek co-ethnic networks where people share the same language and culture, and experience and understand similar difficulties. This emotional support can provide protection from effects of stressors such as discrimination. Therefore, ethnic support may serve as pseudo family support among immigrants in South Korea and protect against the negative effect of perceived discrimination on depressive symptoms.

On the other hand, the results provide strong evidence for the importance of emotional support from a member of the host country for less depressive symptoms among immigrants. Receiving Korean support had direct negative effects on depressive symptoms, however, did not protect the depressive symptoms when immigrants are subjected to discrimination. There is a long-standing belief that Korea is culturally and ethnically a “homogenous” society, but this has been challenged with recent immigrants from different countries. Now immigrants constitute slightly more than 3% of population in South Korea since 1990s [4, 42]. However, many Korean citizens still believe in the myth that Korea shares only one-blood, one language, and one culture [29]. Due to a short immigration history as well as homogeneous context of South Korea, host members are not aware of racial discrimination. Also, perceived cultural threats and national identity can be interpreted as hidden forms of ethnocentrism or xenophobia, which in turn, may cause discrimination against immigrants in South Korea [29]. Thus, discrimination is rampant in Korean society due to nationalistic or xenophobic tendencies among host members. Therefore, in South Korea, host support did not show a protective effect to depressive symptoms when they experience discrimination.

This study has several limitations, which we note briefly. First, these data are cross-sectional. We cannot assess the temporal ordering of perceived discrimination, social support and depressive symptoms. Second, there is a small limitation regarding measurement of family support in that we could not differentiate whether ethnic or host support is from a family member (e.g. husband) or not. Lastly, the sample was withdrawn from the legal immigrant population lists provided by the Ministry of Justice. Thus, the results are more likely to be an underestimation of the total effect of discrimination and social support on depressive symptoms without undocumented immigrants.

Despite these limitations, our study is notable for several reasons. First, this study was significant in that it provided the current status of the relationship between discrimination, social support and mental health for the immigrant population in South Korea, which has been understudied. Moreover, this study examined not only the association between perceived discrimination and mental health among immigrants in South Korea, but also the main and moderating effects of differentiated social support (separate ethnic and host supports) as well as general social support.

In the future, a longitudinal study on immigrants would further strengthen research by enhancing the understanding of the relationship between significant predictors such as perceived discrimination, differentiated social support, and mental health outcomes. Furthermore, comparison of immigrants with Korean nationals would further enrich the knowledge of the immigrants’ health, by providing a baseline comparison and allowing researchers to test for immigrant health advantages and acculturative changes over time.

Our findings have broader policy implications that community-level interventions providing immigrants opportunities to increase social networking with both members in a host country and other immigrants from the same country might be helpful resources for improving mental health among immigrants in South Korea. In the meantime, policy makers should pay attention to raise the awareness of racial discrimination among Korean people as necessary. Highlighting discrimination helps members of a community understand the stress of racial discrimination that it can cause, bringing into focus a perspective that is not often respected. Moreover, although our findings are different from the general health pattern found in the traditional host countries, they add to the new and growing literature of perceived discrimination, social support and immigrant mental health in newer host countries.

## Conclusion

This study aimed to examine the association between perceived discrimination and depressive symptoms, and the role of different social support among immigrant population in one of newer host countries. The results confirmed the association between perceived discrimination and depressive symptoms, along with the moderating effects of ethnic support between the two. Our findings clearly suggest that more public health policy attention should be paid to immigrant population in South Korea. Further studies will enrich the knowledge towards immigrant health.
